# Highly Diverse *Synechococcus* Pigment Types in the Eastern Indian Ocean

**DOI:** 10.3389/fmicb.2022.806390

**Published:** 2022-02-25

**Authors:** Xiaodong Zhang, Shunyan Cheung, Jing Wang, Guicheng Zhang, Yuqiu Wei, Haijiao Liu, Jun Sun, Hongbin Liu

**Affiliations:** ^1^Department of Ocean Science, The Hong Kong University of Science and Technology, Kowloon, Hong Kong SAR, China; ^2^Southern Marine Science and Engineering Guangdong Laboratory, Guangzhou, China; ^3^Research Centre for Indian Ocean Ecosystem, Tianjin University of Science and Technology, Tianjin, China; ^4^State Key Laboratory of Biogeology and Environmental Geology, China University of Geosciences, Wuhan, China; ^5^Hong Kong Branch of Southern Marine Science and Engineering Guangdong Laboratory, The Hong Kong University of Science and Technology, Kowloon, Hong Kong SAR, China; ^6^State Key Laboratory of Marine Pollution, Kowloon, Hong Kong SAR, China

**Keywords:** *Synechococcus*, pigment types, *cpeBA* operon, eastern Indian Ocean, ESTU

## Abstract

Marine picocyanobacteria *Synechococcus* exhibit highly diverse pigment types (PTs) and hence possess great advantage to utilize different spectrum of light effectively and to occupy a wide range of light niches. In this study, we explored the diversity of *Synechococcus* PTs in the eastern Indian Ocean (EIO), surface water of Strait of Malacca (SSM), and coastal waters of Sri Lanka (SSL). All the detected PTs were phycourobilin (PUB) containing PT 3 and showed distinct distribution patterns. Low PUB PT 3a and partial chromatic acclimater PT 3eA dominated in coastal and shallow waters (SSM and SSL). In contrast, high PUB and chromatic acclimaters PT 3dA and PT 3c/3dB were mainly distributed in open ocean (EIO). PT 3dA and PT 3c/3dB occurred at similar depths of the lower euphotic layers but showed distinct distribution pattern that are partially exclusive, indicating that they compete with each other for the same light niche. Interestingly, the newly described PT 3f was detected with high relative abundances at all stations and particularly dominated in the upper euphotic layer in EIO, which was confirmed with PT-specific quantitative polymerase chain reaction (qPCR). The relative abundance of PT 3f was negatively correlated with nutrient level, implying that PT 3f is adapted to oligotrophic waters. Pronounced niche partition of different PTs was observed in the upper and lower layers of euphotic zone in EIO and SSM/SSL. Light, nutrients, and strong stratification may play important roles in the niche partition of different PTs. Further analysis about ecologically significant taxonomic units revealed high diversity within each PT at different locations, which provided insights for understanding specific PT with wide range of niches.

## Introduction

The ubiquitously distributed marine *Synechococcus* are one of the most abundant autotrophic microorganisms in the global ocean (Liu et al., 1997; Veldhuis et al., 1997; Partensky et al., 1999; Zwirglmaier et al., 2008). It was estimated that *Synechococcus* contributed 16.7% of global primary production and play a key role in the global biogeochemical cycles (Partensky et al., 1999; Flombaum et al., 2013). Marine *Synechococcus* form a well-defined cluster 5, which is further divided into three subclusters: 5.1, 5.2, and 5.3 (Fuller et al., 2003; Everroad and Wood, 2012). At least 16 distinct marine *Synechococcus* clades in the subcluster 5.1 were identified and the number will increase if subclusters 5.2 and 5.3 are taken into consideration (Ahlgren and Rocap, 2012; Huang et al., 2012; Mazard et al., 2012; Farrant et al., 2016). *Synechococcus* exhibit diverse physiological characteristics and ecological niches, resulting in wide geographical distributions from subpolar to equatorial regions, including estuarine, coastal, and oligotrophic oceanic waters (Ahlgren and Rocap, 2012; Mazard et al., 2012; Flombaum et al., 2013; Xia et al., 2015, 2017a; Farrant et al., 2016). On the basis of distribution pattern, various *Synechococcus* clades could be further divided into different genetically related subgroups, which were named ecologically significant taxonomic units (ESTUs) (Farrant et al., 2016). Strains within each ESTU that share the same distribution pattern can be linked to specific environmental conditions (Farrant et al., 2016).

Marine *Synechococcus* possess unique accessory pigments in the light-harvesting antennae [phycobilisomes (PBSs)], which consists of a central allophycocyanin core and phycobiliprotein rods as well as associated linkers (Six et al., 2007). On the basis of the composition of phycobiliprotein and the structure of PBSs, *Synechococcus* are divided into three pigment types (PTs) (Six et al., 2007; Humily et al., 2013). For PT 1, the PBS rods only contain phycocyanin (PC) and bind phycocyanobilin (PCB) as the sole chromophore. For PT 2, the rods are comprised of PC and phycoerythrin I (PE-I), which bind both PCB and phycoerythrobilin (PEB). The rods of PT 3 consist of PC, PE-I, and PE-II, binding PCB, PEB, and phycourobilin (PUB). According to the compositional proportion of PUB relative to PEB, PT 3 could be further divided into several subtypes: PT 3a (low PUB), PT 3b (medium PUB), PT 3c (high PUB), and PT 3d (variable PUB) (Six et al., 2007; Humily et al., 2014; Xia et al., 2017b,^2018^; Grébert et al., 2018). *Synechococcus* strains exhibiting the PT 3d phenotype are capable of type IV chromatic acclimation (CA4), a process by which they can modify their PUB/PEB ratio to match the dominant ambient light: blue or green. They could be further divided into two subtypes (CA4-A and CA4-B, hereafter referred to PT 3dA and PT 3dB, respectively) on the basis of gene content, gene organization, and genomic context in the gene island involved in the chromatic acclimation (Humily et al., 2013). However, the Mediterranean Sea strain RCC307 initially thought to be a medium PUB strain (Six et al., 2007) was more recently shown to display a faint chromatic acclimation ability (Humily et al., 2013). Indeed, its PUB:PEB ratio only varied slightly from 0.6 in green light to 0.8 in blue light (Humily et al., 2013). Because of the partial chromatic acclimation and a complete CA4-A gene island, RCC307 was named as PT 3eA (“A” represented the presence of a CA4-A island) to differentiate it from other subtypes (Humily et al., 2013). Recently, a novel high PUB PT 3f with unique evolutionary history was identified on the basis of strain KORDI-100 isolated from surface water of tropical Pacific (Grébert et al., 2018; Xia et al., 2018). Analysis of genome of strain KORDI-100 showed that the intergenic spacer (IGS) sequence in *cpeBA* operon was shorter than other PTs. Gene content and organization of the PBS genomic region of PT 3f were also different from other PTs (Xia et al., 2018). It was reported that PT 3f was globally distributed with relative low abundance but was abundant in some specific regions in the South China Sea, Indian Ocean, and Mediterranean Sea (Grébert et al., 2018; Xia et al., 2018).

Different PTs allow marine *Synechococcus* to harvest distinct regions of the light spectrum more efficiently, and the diversity of PTs plays an important role in geographical distribution and niche segregation of *Synechococcus* (Xia et al., 2017b; Grébert et al., 2018). PT 1 was often observed with high abundance in turbid red light–dominated waters, whereas PT 2 was common in coastal and shelf waters where yellow-green light prevails (Vörös et al., 1998; Wood et al., 1999; Stomp et al., 2007; Xia et al., 2017b,^2018^). PT 3 is the dominant PT in oceanic waters because it contains cells that are able to harvest a wide range of light spectrum, ranging from blue-green to yellow-green (Six et al., 2007; Xia et al., 2017b). Because the light intensity and composition vary with depth or locations, diverse PTs provide a great advantage in occupying different light niches. In addition to light, other environmental factors may also play important roles in niche partitioning of *Synechococcus* with different PTs. The PC-type *Synechococcus* strains were able to outcompete PE-containing strains quickly after adding high concentration of nutrients into *Synechococcus-*enriched water samples according to Hunter-Cevera et al. (2016). PT 3a was found positively related with dissolved inorganic nitrogen (DIN) concentration, whereas PT 3c thrived in low DIN waters and was positively correlated with iron concentration (Grébert et al., 2018). PT 3dB shares similar preference with PT 3c, whereas PT 3dA prefers low-temperature and high-nutrient environments (Grébert et al., 2018; Xia et al., 2018). Similar to PT 3c, PT 3f prefers oligotrophic waters according to Grébert et al. (2018) and Xia et al. (2018).

In this study, we investigated the spatial and vertical distribution of *Synechococcus* PTs in the eastern Indian Ocean (EIO). The Indian Ocean is the third largest ocean in the world, characterized by the strong influence of monsoon and two semi-enclosed basins in the north: the Arabian Sea and the Bay of Bengal (Fine et al., 2008; Rixen et al., 2009). The Indian Ocean experiences prevailing semi-annual currents during the summer and winter monsoon periods (Luyten and Roemmich, 1982; Zhang et al., 2015) including the equatorial undercurrent and the South Java Current (Iskandar et al., 2009; Peng et al., 2015). In the spring and fall inter-monsoon periods, many surface circulations disappear, and the Wyrtki jets (WJs) are the only semi-annual currents present at the equator. Influenced by river discharge, precipitation, and evaporation, salt stratification is formed in the tropical waters in the inter-monsoon (Sprintall and Tomczak, 1992; Patra et al., 2007). Strong stratification suppresses upwelling and mixing of the deep waters, making the eastern equatorial Indian Ocean a typical oligotrophic area (Kumar et al., 2009).

## Materials and Methods

### Sample Collection

**FIGURE 1:**
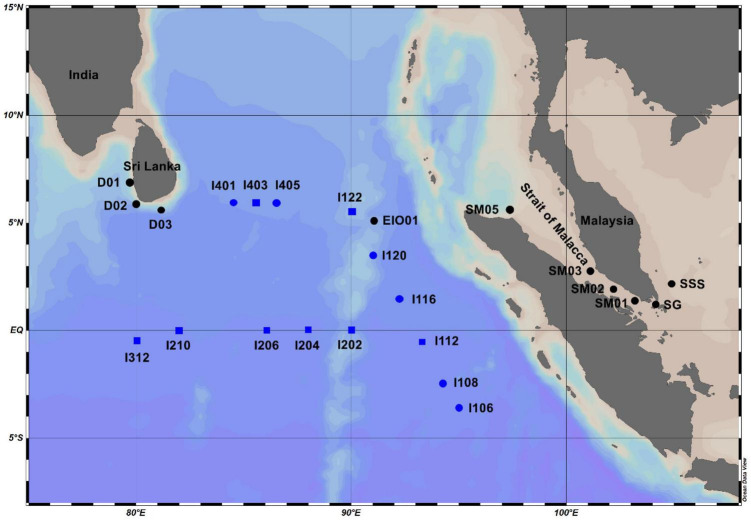
Sampling stations in the eastern Indian Ocean (EIO), coastal waters of Sri Lanka, and the Strait of Malacca. Black dot–labeled stations: samples were collected from surface water in 2018; blue dot–labeled stations: samples were collected from surface water in 2015; blue square–labeled stations: samples were collected from water column (surface: 25, 75, and 150 m) in 2015.

Two cruises were carried out in the EIO onboard R/V Shiyan 3 from March to May in 2015 and 2018. Samples from the open water in EIO were collected in 2015, whereas the samples from Sri Lanka coastal waters (D01, D02, and D03), Strait of Malacca (SG, SM01, SM02, SM03, and SM05), and one station located in Shelf of Sunda (SSS) were collected in 2018 (Figure 1). Water samples from different depths were collected using Niskin bottles (12 L) attached to a conductivity, temperature, and depth rosette multi-sampler (Sea Bird Electronics, Washington, DC, United States). Two liters of water samples were filtered with 0.22-μm PC membranes (Millipore, Eschborn, Germany) under low pressure to collect the DNA samples. The membranes were frozen in liquid nitrogen immediately after filtration. For chlorophyll *a*, 1 L of water samples were filtered with glass microfiber filters (GF/F) filters, and the filters were then frozen in liquid nitrogen immediately on board. For nutrients concentration analysis, 100 ml of water samples were frozen at −20°C after filtering through acid-cleaned 0.45-μm acetate cellulose filters. Water samples (1.8 ml) for abundance measurement by flow cytometer were pre-filtered with 20-μm mesh, preserved with paraformaldehyde (1% final concentration) and frozen in liquid nitrogen. All the samples were stored at −80°C in the laboratory after the cruise until analysis except the samples for nutrients which were stored in −20°C. The monthly diffuse attenuation coefficients at 490 nm (Kd490) which was an indicator of water turbidity in sampling stations, were retrieved from the NOAA database^1^.

### Chlorophyll *a*, Nutrients Measurement, and Flow Cytometer Analysis

Chlorophyll *a* was extracted with 90% acetone for 18–24 h at 4°C in the dark and measured by a Turner Designs Trilogy Laboratory Fluorometer (Turner Designs, San Jose, CA, United States) with CHL-A NA model following the work of Welschmeyer (1994). Dissolved inorganic nutrients including nitrate, nitrite, ammonium, phosphate, and silicate were analyzed with a Technicon AA3 Auto-Analyzer (Bran + Luebbe, Hamburg, Germany) on the basis of the standard procedures (Hansen and Koroleff, 2007). *Synechococcus* cells were enumerated using a flow cytometer (Becton-Dickinson Accuri C6) equipped with a 488-nm laser. A total volume of 198 μl of samples was analyzed with a flow rate of 66 μl/min for 3 min, and 2-μm fluorescent beads (Polysciences, Warrington, PA, United States) were added as the internal standard (Olson et al., 1993).

### DNA Extraction, Polymerase Chain Reaction Amplification, and Sequencing

DNA was extracted using the enzyme/phenol-chloroform protocol described previously (Riemann et al., 2000). The primers peBF: 5′-adaptor-barcode-GACCTACATCGCWCTGGGYG-3′ and peAR (5′-CCMACAACCARGCAGTAGTT-3′) were applied to amplify the *cpeBA* operon of *Synechococcus* (Xia et al., 2018). The polymerase chain reaction (PCR) was carried out with triplicates in 25-μl mixture including 2.5 of μl 10 × buffer, 1 μl of MgCl_2_, 1 μl of deoxyribonucleoside triphosphate, (dNTPs, 2.5 mM), 0.1 μl of Invitrogen Platinum Taq DNA polymerase (Life Technologies, Carlsbad, CA, United States) (5 U), 1 μl of DNA template, and 0.5 μl of solution (10 μM) of each primer. The PCR program followed that in the work of Xia et al. (2018), and PCR products were stained with SYBR green and visualized on 1% agarose gel after electrophoresis.

### Sequencing Analysis of *cpeBA* Operon and Statistical Analysis

Sequencing of the PCR products was conducted using an Ion-Torrent PGM system in Nova Company, and the data were analyzed by using software Mothur (Schloss et al., 2009). Quality control was conducted by trimming the low-quality sequences with incorrect length or containing ambiguous bases as well as homopolymers longer than 10 bp. Only reads with an average quality score above 25 were kept for further analysis. The chimeric sequences were then removed using chimera.uchime command, and the sequences were aligned using align.seqs command. One thousand three hundred reads were subsampled from each sample due to the limitation of computer capability. The sequences were clustered into operational taxonomic units (OTUs) at 95% DNA similarity. An excel file containing sequences number of each OTU was generated using Mothur’s make.shared routine. Maximum likelihood phylogenetic tree was constructed with MEGA 7 (Kumar et al., 2016) with 1,000 bootstraps. The most similar reference sequences were retrieved from the national center for biotechnology information (NCBI) database. The statistical analysis of canonical correspondence analysis (CCA), non-metric multidimensional scaling (NMDS), and Spearman analysis were conducted by Past software (Hammer et al., 2001). For each PT, hierarchical clustering was performed using a similarity profile (SIMPROF) test (method.distance = Euclidean) of the package “clustsig” in R (Whitaker and Christman, 2010) to group OTUs with similar distributional patterns (*P*-value < 0.05) into ESTUs. All the sequences in this study were submitted to Sequence Read Archive database in NCBI with BioProject accession number PRJNA768338.

### Quantitative Analysis of PT 3a, PT 3dA, and PT 3f

The primers for quantitative PCR (qPCR) analysis of different PTs of *Synechococcus* were designed on Primer3Plus online,^2^ which were based on the top OTU of PT 3a, PT 3dA, and PT 3f in the sequencing dataset. The degeneracy of the primers was applied to cover more OTUs of each PT. The information of new primer sets was shown in Supplementary Table 1.

The top OTUs of PT 3a, PT 3dA, PT 3c/3dB, PT 3eA, and PT 3f were synthesized in GeneRay company (Xiamen, China) and were used as the standard DNA for qPCR or used in test of cross-sensitivity. Triplicated qPCR reactions targeting the *Synechococcus cpeBA* operon were performed by Roche LightCycler 480 Realtime PCR System in a 10-μl mixture containing 1 × LightCycler^®^ 480 SYBR^®^ Green I Master, 0.5 μM primers pairs, and 1-μl DNA templates. The thermal cycle of the qPCR started with 5-min denaturation at 95^°^C, followed by 50 cycles each at 95^°^C for 15 s, 55^°^C for 45 s, and 72^°^C for 15 s, with single-signal acquisition at the end of each cycle. Amplification specificity was confirmed *via* the melting curve.

## Results

### Hydrographic Conditions and Environmental Factors in Sampling Area

Warm and salty water was observed in the surface of EIO. The average surface seawater temperature and surface seawater salinity in the EIO were 29.93^°^C and 34.04, respectively. SSL and SSM were warmer and less salty compared with the basin of EIO water (Supplementary Figure 1). Significant thermoclines were identified between 50 and 100 m depth in EIO, indicating a pronounced stratification (Supplementary Figure 2). The mixed layer depth ranged from 44 m (I106) to 89 m (I405) with the average depth of 64.5 m at the sampling stations. However, in all the stations where the samples of vertical column were collected, the mixed layers were less than 75 m. DIN and phosphate were depleted in the surface water of EIO, ranging from 0.191 to 0.494 and 0.013 to 0.077 μmol/L, respectively (Supplementary Figure 1). Both DIN and phosphate concentration were remarkably higher in the lower euphotic layers than that in the upper euphotic layers, and maximum values were observed in 75 m, generally (Supplementary Figure 2). The nitogen/phosphate (N/P) ratio ranged from 0.42 (surface water of I112) to 45 (surface water of I122). However, N/P ratio in 90% of the samples was less than 12, indicating N-limitation in EIO. The average concentrations of DIN and phosphate, as well as N/P ratio, in the surface water of SSM and SSL were remarkably higher than that of EIO.

### *Synechococcus* Abundance

Distribution of *Synechococcus* was generally restricted to the upper layers of the water column in EIO, with the maximum abundance observed in the surface layer of all sampling stations (Supplementary Figure 2), ranging from 2.37 × 10^3^ to 1.82 × 10^4^ cells/ml^–1^ and decreasing with depth. In SSM and SSL, the abundance of *Synechococcus* ranged from 1.14 × 10^4^ to 6.78 × 10^4^ cells/ml^–1^ and 1.38 × 10^4^ cells/ml^–1^ to 6.55 × 10^4^ cells/ml^–1^, respectively, with only PE-type *Synechococcus* were observed in these sampling stations.

### Diversity and Distribution of *Synechococcus* Pigment Types

**FIGURE 2:**
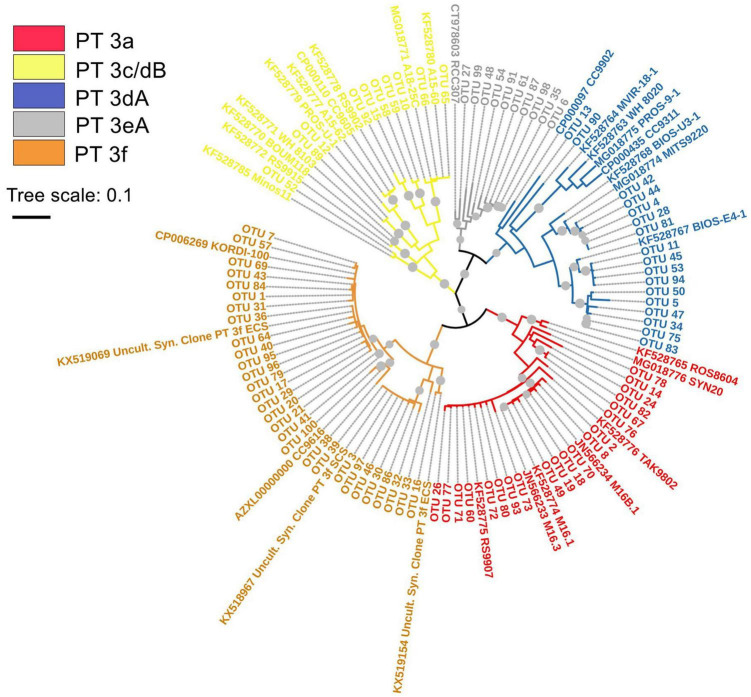
Maximum likelihood phylogenetic tree of *cpeBA* operon sequences of abundant operational taxonomic units (OTUs) detected in eastern Indian Ocean (EIO), surface water of coastal waters of Sri Lanka (SSL), and the Strait of Malacca (SSM) with bootstrap of 1,000 replicates. The bootstrap values higher than 50% are displayed as gray circles. The abbreviation of Uncult.Syn. Clone PT 3f ECS and SCS represents the uncultured *Synechococcus* clone sequence of PT 3f detected in East China Sea (ECS) and South China Sea (SCS), respectively.

In this study, no PT 2 *Synechococcus* were detected in both EIO and SSL/SSM. One combination of two subtypes (3c/3dB) and four others (3a, 3dA, 3eA, and 3f) of PT 3 were observed and formed five separate clusters in the phylogenetic tree (Figure 2). The weak chromatic acclimater PT 3eA was the most phylogenetically close with PT 3dA. For PT 3f, three subclusters were well separated in the phylogenetic tree, indicating potential high genetic diversity within this PT group.

**FIGURE 3:**
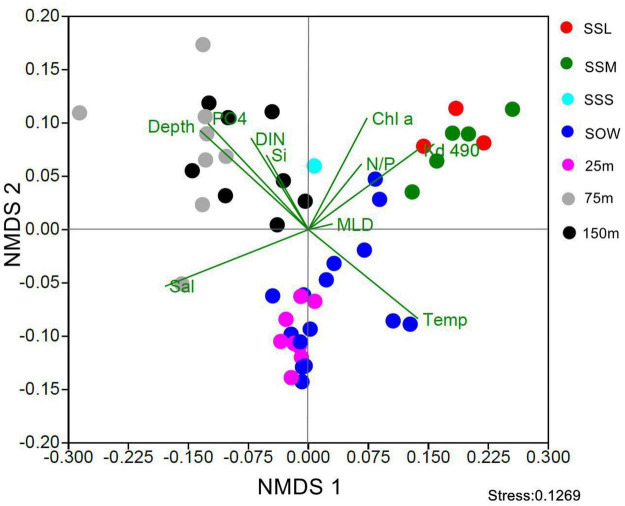
NMDS analysis of community of *Synechococcus* pigment types in different depths and sampling stations (SSL, surface water of Sri Lanka coastal waters; SSM, surface water of Strait of Malacca; SSS, Surface water of station Shelf of Sunda; SOW, surface water of open water in EIO; Temp, temperature; Sal, salinity; DIN, dissolved organic nitrogen; PO4, phosphate; N/P, N/P ratio; Si, silicate; Chl *a*, chlorophyll *a*; MLD, mixed layer depth; Kd490, the diffuse attenuation coefficients at 490 nm).

NMDS analysis showed that the community structure of *Synechococcus* PT was distinct between different locations and layers by forming three well-separated groups (Figure 3). The first group consisted of samples collected from upper euphotic layers in EIO including the surface (0 or 5 m) and 25 m depth; the second group was composed of samples collected from lower euphotic layers in EIO (75 and 150 m); whereas the samples collected from SSM and SSL formed the third group.

**FIGURE 4:**
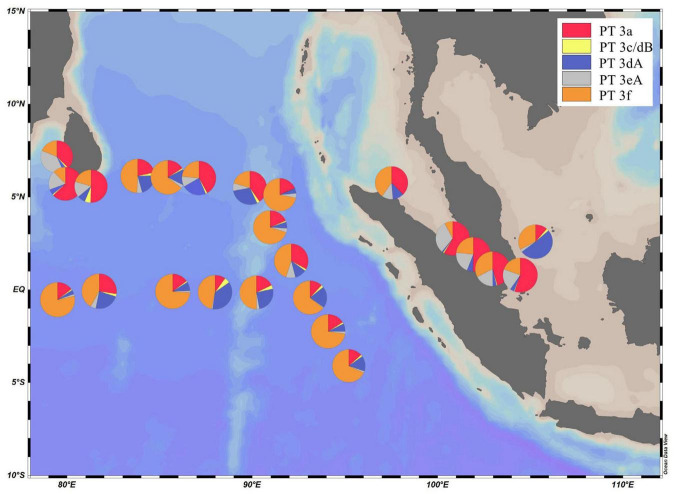
Spatial distribution *Synechococcus* pigment types in the surface water of eastern Indian Ocean (EIO), coastal waters of Sri Lanka and the Strait of Malacca.

**FIGURE 5:**
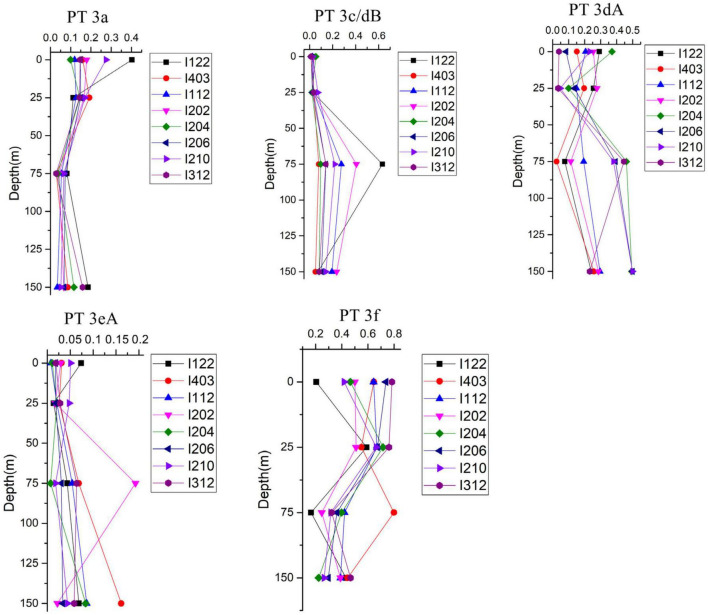
Vertical distribution of relative abundance of different *Synechococcus* pigment types in eastern Indian Ocean (EIO).

**FIGURE 6:**
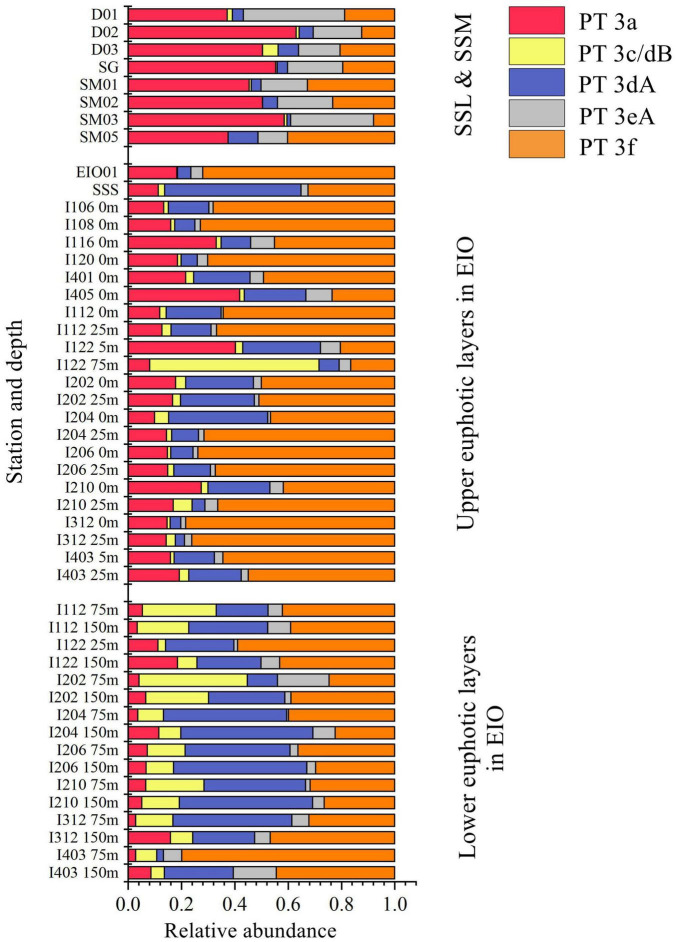
Community structure of *Synechococcus* pigment types in different depths and sampling sites eastern Indian Ocean (EIO), Sri Lanka coastal waters, and Strait of Malacca (SSL, surface water of Sri Lanka coastal waters; SSM, surface water of Strait of Malacca; SSS, Surface water of station Shelf of Sunda).

Low PUB PT 3a was the dominant PT in SSL and SSM with the average percentage of 50.1 and 49.5%, respectively (Figure 4). In comparison, the relative abundance of PT 3a was significantly lower in the surface water of EIO than in SSL/SSM (Figure 4). PT 3a was much more abundant in the upper (19% in average) than the lower euphotic layers (7.4% in average) in EIO (Figures 4–6). Both PT 3dA and PT 3c/3dB were significantly less abundant than other PTs in SSL and SSM (Figures 4, 6), and PT 3c/3dB were even undetectable at SM02 and SM05. In EIO, the two chromatic acclimaters showed different vertical distribution patterns. In the upper euphotic layers, PT 3c/3dB were minor groups, whereas PT 3dA was abundant at certain stations (Figures 4, 6). The relative abundance of PT 3c/3dB reached its peak at 75 m and then declined at 150 m, whereas no obvious vertical distribution pattern of PT 3dA was observed (Figures 5, 6). Similar with PT 3a, relative abundance of PT 3eA was significantly higher in SSL and SSM than in EIO as well as the station SSS (Figures 4, 6). PT 3eA contributed 11–38% of biomass in SSL and SSM, whereas, in surface water of EIO, the average relative abundance of PT 3eA was only 3.9%. PT 3eA was the minor group in both the upper and lower euphotic layers in EIO, although the relative abundance increased slightly with depth generally (Figures 5, 6). The newly described PT 3f was abundant generally across sampling stations. PT 3f dominated in the surface water of EIO (Figures 4, 6), with the average percentage of 58.6%, but declined to 37.1% in the lower euphotic layers. In SSL and SSM, the PT 3f was also abundant and the relative abundance was 17.2 and 26%, respectively. In SSS and SM05, which were also oligotrophic stations located at Sunda Shelf and the end of Strait of Malacca, the relative abundance of PT 3f reached 32.5 and 40.2%, respectively. The distribution pattern in surface water of sampling area indicated the oligotrophic preference of PT 3f.

**FIGURE 7:**
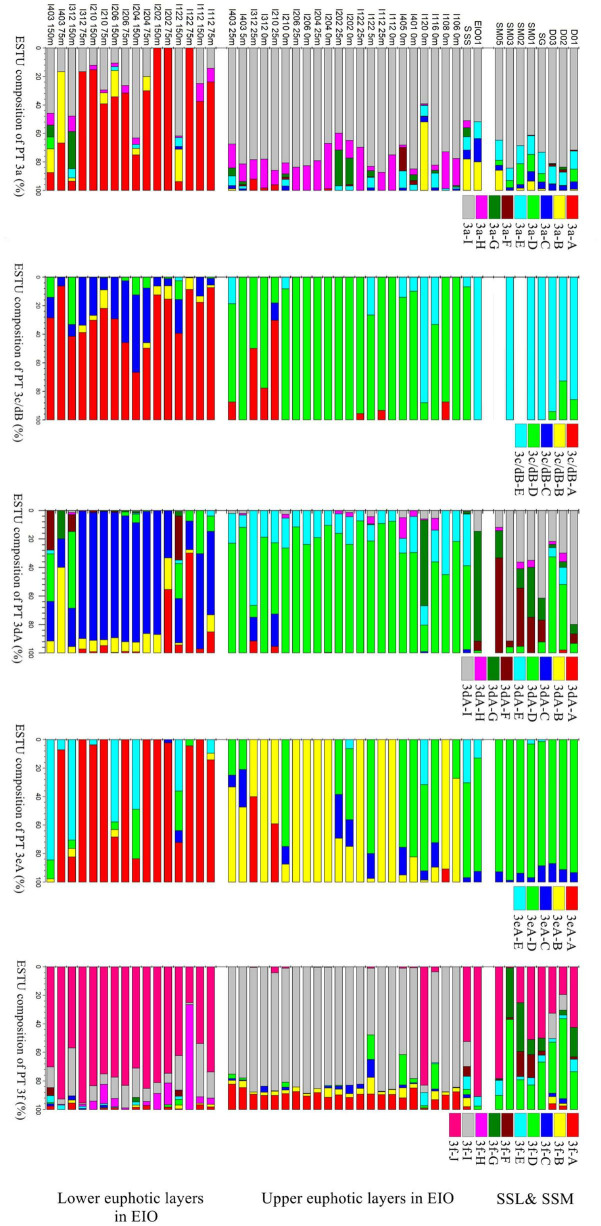
Divergence of different ecologically significant taxonomic units (ESTUs) within each PT in surface water of coastal waters of Sri Lanka (SSL), the Strait of Malacca (SSM), and the upper and lower euphotic layers in eastern Indian Ocean (EIO).

To further explore the ecological divergence within each PTs, the ESTUs clustering OTUs with different distribution pattern were applied (Figure 7). Five to ten ESTUs of different PTs were successfully constructed. For each PT, the upper and lower layers of euphotic zone in EIO and SSL/SSM were occupied by different ESTUs (Figure 7), indicating that each PT was phylogenetically and ecologically divergent. For example, relative abundant PT 3dA was observed in both lower euphotic zone and some stations in the upper euphotic zone, indicating their ability to adapt different light conditions. However, the ESTU composition in the upper euphotic layers, lower euphotic layers, and SSL/SSM was completely different. In the upper layers of euphotic zone in EIO, PT 3dA was mainly composed of ESTU 3dA-D and 3dA-E, whereas the lower layers of euphotic zone were mainly occupied by ESTU 3dA-C. In SSL and SSM, ESTU 3dA-F and 3dA-I were the main groups (Figure 7). The divergence of different ESTUs suggested the niche partition within PT 3dA. Similarly, pronounced divergence of ESTUs within other PTs at different locations was also observed (Figure 7), indicating the ecological divergence of each PT and the potential of *Synechococcus* to occupy wider ecological niches.

### Relationship Between Environmental Factors and Distribution of *Synechococcus* Pigment Types

The spearman and CCA analysis showed that both the physical and chemical parameters may play important role in shaping the distribution of various PTs (Supplementary Figures 3, 4). Spearman analysis showed that PT 3a was negatively correlated with depth, salinity, DIN, and phosphate but positively correlated with temperature; PT 3eA was negatively correlated with salinity, whereas the correlations with other parameters were not significant. However, after eliminating possible effects of light and depth, the CCA analysis based on the samples from the surface water only showed positive correlations between PT 3a and PT 3eA and DIN and turbidity (represented by Kd490, the diffuse attenuation coefficients at 490 nm) (Supplementary Figure 4). DIN and turbidity may play key roles in shaping the surface distribution of PT 3a and PT 3eA. The CCA analysis based on samples collected from water column in eight stations in EIO suggested that PT 3a and PT 3eA possessed opposing preference of depth, DIN, and phosphate in vertical profile (Supplementary Figure 4). For both PT 3c/3dB and 3dA, the vertical distribution was negatively correlated with temperature and positively correlated with depth, salinity, and nutrients (Supplementary Figure 4). PT 3f showed no specific preference of temperature, salinity, phosphate, and depth but had a negative relationship with DIN in both surface and vertical profile (Supplementary Figures 3, 4). Turbidity was negatively correlated with PT 3c/3dB, PT 3dA, and PT 3f (Supplementary Figure 4), indicating a key role of light spectrum in the distribution of these high PUB/PEB PTs. Both the CCA of surface water samples and the Spearman correlation analysis showed that PT 3f was strongly negatively related with nutrients, suggesting the preference of PT 3f to oligotrophic waters (Supplementary Figures 3, 4).

### Quantification of PT 3a, PT 3dA, and PT 3f

The abundance of PT 3a, PT 3dA, and PT 3f in EIO were quantified with qPCR using newly designed primer sets. The specificity of each primer set and the cross-reactivity were tested before quantifying the abundance of three PTs in surface and 25 m in open ocean, and the results were summarized in Supplementary Table 2. For primer sets targeting PT 3f, the difference of C_T_ values obtained from non-targeting templates and PT 3f template were at least 8.62, suggesting a low amplification efficiency of other PTs. Primer sets targeting PT 3dA failed to detect PT 3c/3dB and PT 3f, and the C_T_ values obtained from PT 3a and PT 3eA templates were similar with non-template control. For primer sets targeting PT 3a, the differences of C_T_ values obtained for targeting and non-targeting template ranged from 6.29 to 21.16 (Supplementary Table 2), suggesting that the copies number detected from PT 3a template were two to seven orders of magnitude higher than non-targeting templates. The differences of C_T_ values between targeting and non-targeting templates (Supplementary Table 2) indicated that the newly designed primer sets were specific, and the existing of non-targeting PTs will not affect the estimation of the abundance of the targeting PTs significantly. For PT 3c/dB, the sequence of *cpeBA* operon was significantly diverse, and it was very difficult to design efficient and specific primers targeting PT 3c/dB. The *cpeBA* operon gene copies of PT 3eA were not measured because PT 3eA was a minor group in the open ocean. The quantification only focused on samples collected from surface water (0 or 5 m) and 25-m-deep water in EIO as it was difficult to detect the *cpeBA* operon genes of *Synechococcus* from 75 and 150 m due to their low abundance. The relative abundance of three PTs and gene copies of *cpeBA* operon were shown in Supplementary Figure 5. On the basis of quantified analysis of *cpeBA* operon gene copies numbers, PT 3f was much more abundant than PT 3a and PT 3dA in surface and 25-m-deep waters. The average concentrations of *cpeBA* operon gene copies for PT 3f, PT 3dA, and PT 3a in the tested samples were 1.28 × 10^4^, 9.01 × 10^2^, and 7.84 × 10^3^ copies/ml, respectively, which matched well with the results of sequencing.

## Discussion

### Wide Distribution of PT 3eA and PT 3f

Both PT 3eA and PT 3f were rarely reported. The representative strain of PT 3eA, RCC307, was initially isolated from the subsurface water of Mediterranean Sea and identified as PT 3b due to its medium PUB content (Six et al., 2007). Humily et al. (2013) examined strain RCC 307 and suggested that it possessed a CA4-A gene island and weak ability of chromatic acclimation. To distinguish these strains with typical PT 3b and PT 3dA strains, Humily and colleagues classified strain RCC 307 as PT 3eA (Humily et al., 2013) and multiple names were applied to describe RCC 307 in the subsequent studies, including PT 3b (Six et al., 2007; Xia et al., 2018), PT 3eA or 3e (Humily et al., 2013, 2014; Grébert et al., 2021a,b), PT 3dA (Grébert, 2017; Xia et al., 2017b; Chen et al., 2021), and asterisk-highlighted PT 3b, 3e, or 3dA to indicate its uniqueness (Xia et al., 2018; Sanfilippo et al., 2019). In this study, we used PT 3eA following the work of Humily et al. (2013) to describe the subtype of RCC 307 to differentiate it from typical PT 3b and PT 3dA.

Previous studies showed that it was very difficult to clearly separate RCC307 from PT 3dA based on phylogenetic trees of both *Fci* sequences and *cpeBA* operon (Humily et al., 2013; Xia et al., 2017b,^2018^). However, Grébert et al. (2021a,b) indicated clear divergence between RCC 307 and PT 3dA based on *mpeBA* operon. Our results also showed that multiple RCC 307 closely related strains (represented by OTUs) fell into PT 3eA clade that was separated from PT 3dA in phylogenetic tree of *cpeBA* operon (Figure 2), indicating the phylogenetic divergence of PBS rods gene region between PT 3eA and PT 3dA. These RCC 307 closely related strains were detected in every samples and abundant in SSM and SSL, suggesting their wide distribution. A recent study based on the analysis combining metagenomics, metatranscriptomics, and amplicon approaches also indicated that *Synechococcus* strains phylogenetically closely related with RCC307 were widely distributed and abundant in South China Sea (Chen et al., 2021). However, it should be noted that, although phylogenetic divergence between RCC307 and PT 3dA was observed in both our results and that of Grébert et al. (2021a,b) on the basis of *cpeBA* and *mpeBA* phylogeny, PT 3eA could not be classified into a complete new subtype as no significant difference of gene content and organization in PBS rod region between RCC307 and PT 3dA was observed. Moreover, these widely distributed and abundant strains were possibly from subcluster 5.3 and possess a CA4-A island that could display typical 3dA phenotype chromatic acclimation. Because RCC307 is the only representative strain of PT 3eA and knowledge about its physiological and ecological characteristics is limited, further studies focusing on isolated strains and field-based exploration are necessary.

The high PUB PT 3f is a newly described PT on the basis of strain KORDI-100 isolated from surface water of tropical Pacific (Mahmoud et al., 2017; Xia et al., 2017b,^2018^; Grébert et al., 2018). Analysis of strain KORDI-100 genome showed that the IGS sequence in *cpeBA* operon and gene organization of the PBS was different from other PTs (Xia et al., 2018). Until now, two strains of *Synechococcus* (KORDI-100 and CC9616) had been classified as PT 3f (Mahmoud et al., 2017; Xia et al., 2017b,^2018^; Grébert et al., 2018). Phylogenetic analysis of strain KORDI-100 and CC9616 showed that these two strains belonged to rarely detected clade UC-A and XX (previous EnvC) (Grébert et al., 2018; Xia et al., 2018). On the basis of amplicon and metagenomics analysis, it was reported that PT 3f was closely related with clades II, III, WPC1, and XX (Xia et al., 2018; Grébert et al., 2018). Our results also showed that PT 3f was highly phylogenetically diverse, indicating that multiple clades, not only clade XX and clade UC-A, may possess PT 3f.

Grébert et al. (2018) indicated that PT 3f was globally distributed. Although the relative abundance of PT 3f was lower than other PTs in general, they were abundant in some areas of Indian Ocean and Mediterranean based on metagenomic analysis of data from *Tara* Oceans (Grébert et al., 2018). Xia et al. (2018) also clarified that the relative abundance of PT 3f increased from coastal area (less than 5%) to basin (∼50%) in South China Sea. In mesotrophic area of East China Sea, the relative abundance of PT 3f was also significantly lower than that in the oligotrophic basin of South China Sea (Xia et al., 2018). In our results, PT 3f was dominant in oligotrophic upper layers of euphotic zone in EIO and was further confirmed by qPCR method, which indicated their preference to oligotrophic conditions. Because of the significant portion of relative abundance and wide distribution in oligotrophic waters of EIO, PT 3f may contribute a large proportion to the primary production of *Synechococcus* in this region. Our results of PT 3f were different from the general global distribution pattern revealed by Grébert et al. (2018), which was possibly caused by difference of sampling areas, seasonal variation, and use of a metagenomic approach devoid of amplification biases. Because of wide distribution and significant abundance, further physiological and ecological studies about PT 3f should be conducted in future.

### Strong Stratification Influenced Vertical Distribution of Different Pigment Types

NMDS analysis showed that the community structure of *Synechococcus* PTs in the upper and lower euphotic layers was completely different (Figure 3). However, we could not separate the community structure of surface and 25-m-deep layer. Similarly, the community structure of *Synechococcus* PTs of 75 and 150 m was also merged with each other. The CCA analysis of surface and vertical distribution of *Synechococcus* PTs (Supplementary Figure 4) showed that the mixed layer depth had a little influence on the communities of *Synechococcus* PTs in surface water but may influence the vertical profile of *Synechococcus* PTs. In the eight stations where vertical profile samples were collected, the mixed layer depth located between 25 and 75 m. The niche segregation in water column was likely promoted by environmental factors shaped by pronounced stratification. It was reported that the vertical distribution of high- and low-light ecotypes of *Prochlorococcus* was determined by light and temperature variations shaped by water column stratification (West and Scanlan, 1999). It is likely that strong stratification suppressed the mixing between upper and lower layers of euphotic zone and exacerbated the vertical niche separation, resulting to the niche partition of different *Synechococcus* PTs in EIO.

### Niche Partition and Divergence of Pigment Types

PT 2 was mainly observed in coastal shelf regions or transition zone with intermediate optical properties (Grébert et al., 2018); however, no PT 2 was detected in SSL and SSM in this study. The monthly Kd490 data (ranging from 0.033 to 0.183 m^–1^) and detection of abundant *Prochlorococcus* in SSL and SSM (Wang et al., 2015; Wei et al., 2020) indicated that they were not typical shallow coastal waters and were strongly influenced by oceanic waters. The relative abundances of PT 3a showed a declining trend from SSL/SSM to the upper euphotic layers and then to the lower euphotic layers in EIO, with the average percentage of 49.7, 19.0, and 6.6%, respectively. Compared with PT 2, PT 3a could benefit from a wider PAR spectra, extending from blue-green to yellow-green (Six et al., 2007; Xia et al., 2017b), which could allow them to adapt to multiple environments. It was reported that PT 3a was positively related with inorganic nitrogen and turbidity (represented by Kd490) although the influence of turbidity was not as strong as that in PT 2 (Xia et al., 2018). In our results, the CCA analysis of surface water samples showed that PT 3a was positively influenced by DIN and turbidity (Kd490) (Supplementary Figure 4), indicating the key role of light and nutrients on the distribution of PT 3a in surface water. In general, the relative abundance of PT 3a declined with water depth. The low PUB/PEB ratio may not provide powerful advantage when competing with high PUB or chromatic acclimaters to utilize the light (mainly blue light) in the lower euphotic layers. Although the nutrients concentration increased with depth, the light and depth may play a negative role for PT 3a in the lower layers. PT 3eA was a minor group in both the upper and lower euphotic layers in EIO, whereas it was relatively abundant in SSL/SSM. The CCA analysis based on surface water samples showed that PT 3eA was strongly influenced by turbidity (Kd490). The relative abundance of PT 3eA increased slightly with depth (Figure 5), indicating that the chromatic acclimation ability provided advantage over PT 3a in the lower euphotic layers. The high PUB PT 3f was the predominant PT in EIO, and it was abundant in SSL/SSM. They showed great advantage in oligotrophic waters over other PTs. Both PT 3c/3dB and PT 3dA were rarely detected in SSL/SSM. In water column of oceanic waters, the two PTs displayed distinct vertical distribution patterns and were exclusive geographically in the lower euphotic layers especially in 75 m (Figures 5, 6), indicating that they competed for the same light niche. Relative abundance of PT 3c/3dB increased in the lower euphotic layers as compared with the upper euphotic layers in EIO, suggesting that high PUB content or PUB/PEB ratio variation could help *Synechococcus* absorb blue light efficiently in the lower euphotic layers.

Relatively abundant PT 3dA was also observed in the upper euphotic layers at some stations in EIO, mainly composed of four OTUs which were rarely observed in the lower euphotic layers, suggesting the niche partitioning of different subgroups within PT 3dA in water column. We applied the concept of ESTUs to delineate the niche partitioning of various potential subgroups within each PT. Farrant et al. (2016) analyzed the biogeography of ESTUs globally within different clades of picocyanobacteria and unveiled the significantly overlooked ecological diversity based on *petB* gene. For *cpeBA* operon of *Synechococcus*, niche partitioning of highly diverse ESTUs was also observed within each PT in the upper and lower euphotic layers of EIO and SSL/SSM (Figure 7). The phylogenetic divergence of ESTUs within one PT was most likely due to the niche partitioning of various clades possessing the given PT. It was estimated that the divergence of PBS resulted from lateral gene transfer events, whereas PBS rods gene region apparently possessed an evolution history different from that of the core genome (Six et al., 2007; Grébert et al., 2021a). Phylogeny of PBS rods gene region showed that different clades that possessed one same PT could not be distinguished well in previous studies (Xia et al., 2017b,^2018^; Grébert et al., 2018). However, Grébert et al. (2021a) indicated that, for a given PT, the tree topology of *mpeBA* phylogeny actually resembled the topology based on the core gene *petB* although some exceptions existed due to inter-clade lateral gene transfers, suggesting the divergence of PBS rods gene region between different clades possessing the same PT. In this study, the niche partitioning of various ESTUs was possibly resulted from potential divergence between different phylogenetic clades that possessed the same PT but occupied different habitats.

## Conclusion

In this study, distinct vertical and horizontal distribution patterns of PUB-containing *Synechococcus*, including PT 3a, PT 3dA, PT 3eA, PT 3f, and a combination of PT 3c/3dB were detected from EIO and SSM/SSL. Among them, PT 3a and PT 3eA dominated in SSM/SSL. High PUB PT and chromatic acclimaters PT 3dA and PT 3c/3dB were mainly distributed in open ocean (EIO) and exhibited distinct vertical distribution patterns that are partially exclusive, indicating that they compete for the same niche in water column. The newly described PT 3f was widely distributed and dominated in the upper euphotic layers in EIO. Highly ecological divergence ESTUs within each PT were observed in both the upper and lower euphotic layers of EIO, as well as SSM/SSL. The divergence of ESTUs within each PT may help them to occupy wider ecological niches.

## Data Availability Statement

The datasets presented in this study can be found in online repositories. The names of the repository/repositories and accession number(s) can be found below: https://www.ncbi.nlm.nih.gov/, PRJNA768338.

## Author Contributions

XZ wrote the manuscript. SC gave suggestive comments on this draft. JW and HjL attended the cruises and collected the samples. GZ and YW were responsible for the measurements of nutrients concentration and *Synechococcus* abundance, respectively. JS and HbL designed the sampling scheme and revised the manuscript. All authors read and approved the final manuscript to be published.

## Conflict of Interest

The authors declare that the research was conducted in the absence of any commercial or financial relationships that could be construed as a potential conflict of interest.

## Publisher’s Note

All claims expressed in this article are solely those of the authors and do not necessarily represent those of their affiliated organizations, or those of the publisher, the editors and the reviewers. Any product that may be evaluated in this article, or claim that may be made by its manufacturer, is not guaranteed or endorsed by the publisher.

## Footnotes

1
https://oceancolor.gsfc.nasa.gov/l3/order/

2
https://www.bioinformatics.nl/cgi-bin/primer3plus/primer3plus.cgi
