# Supplementary material for: Highly Diverse Synechococcus Pigment Types in the Eastern Indian Ocean
Source: Front Microbiol. 2022 Feb 25;13:806390. doi: 10.3389/fmicb.2022.806390 (PMC8914260; doi:10.3389/fmicb.2022.806390)
Supplement: Supplementary file 1 [file Data_Sheet_1.pdf]

Table S1. Information of primer sets targeting *cpeBA* operon of PT 3a, PT 3dA and PT 3f

Table S2. Cross reactivity of different primer sets as indicated by the threshold cycle number (C_T) values obtained with targeting and non-targeting templates. ND: not detected

Fig. S1. The distribution of temperature, salinity, dissolved inorganic nitrogen (DIN), phosphate, silicate, chlorophyll *a* concentration, Kd490 (the diffuse attenuation coefficients at 490 nm), and *Synechococcus* cell abundance in the surface water of eatern Indian Ocean (EIO), coastal waters of Sri Lanka and Strait of Malacca.

Fig. S2. The vertical profile of temperature, salinity, dissolved inorganic nitrogen (DIN), phosphate and *Synechococcus* cell abundance at sampling stations in eatern Indian Ocean (EIO).

Fig. S3. Correlation analysis between *Synechococcus* pigment types and environmental parameters based on Spearman analysis. The circle scale shows the degree of postive correlation (blue) or negative correlation (red) between two variables and a corss means the correlation was not significant ($p>0.05$). Abbreviation in the figures: Temp, temperature; Sal, salinity; DIN, dissolved organic notrogen; PO4, phosphate; N/P, nitrogen/phosphate ratio; Si, silicate; Chl *a*, chlorophyll *a*; MLD, mixed layer depth; Kd490, the diffuse attenuation coefficients at 490 nm.

Fig. S4. CCA analysis of community of *Synechococcus* pigment types in surface waters and water column of sampling stations. Abbreviation in the figures: Temp, temperature; Sal, salinity; DIN, dissolved organic notrogen; PO4, phosphate; N/P, nitrogen/phosphate ratio; Si, silicate; Chl *a*, chlorophyll *a*; MLD, mixed layer depth; Kd 490, the diffuse attenuation coefficients at 490 nm.

Fig. S5. Comparison of relative abundance and *cpeBA* operon gene copies concentration of PT 3a, PT 3dA and PT 3f in EIO. A: The *cpeBA* operon gene copies concentration (copies/ml, Log10 transformed); B: Relative abundance of targeting OTUs.

Pigment types	Primer sets	Sequence	Length of targeting fragments
PT3dA	PT3dA-F	CTGAACGGYTTCTCGCTTTC	215bp
	PT3dA-R	AGCTTTTCAGCAGCTTCMAG	
PT3a	PT3a-F	CCTCAACRCMAACCTYCATC	247bp
	PT3a-R	GGGGTACTTGTTGAAGCAR	
PT3f	PT3f-F	ATCGGCTGAGTCMATTCGTT	256bp
	PT3f-R	CTTCCTTGGTGACRGCATCM	

Table S1. Information of primer sets targeting *cpeBA* operon of PT3a, PT3dA and PT3f

Primer Set	Template					Non-template controls
	OTU2, PT3a	OTU6, PT3b	OTU5, PT3dA	OTU10, PT3c/3dB	OTU1, PT3f	
PT3a_1F/1R	20.05	29.57	41.21	26.34	35.39	43.14 or ND
PT3dA_1F/1R	47.17	37.38	15.78	ND	ND	47.74 or ND
PT3f_1F/1R	24.59	29.25	40.44	26.97	15.97	39.62 or ND

Table S2. Cross reactivity of different primer sets as indicated by the threshold cycle number (C_T) values obtained with targeting and non-targeting templates. ND: not detected

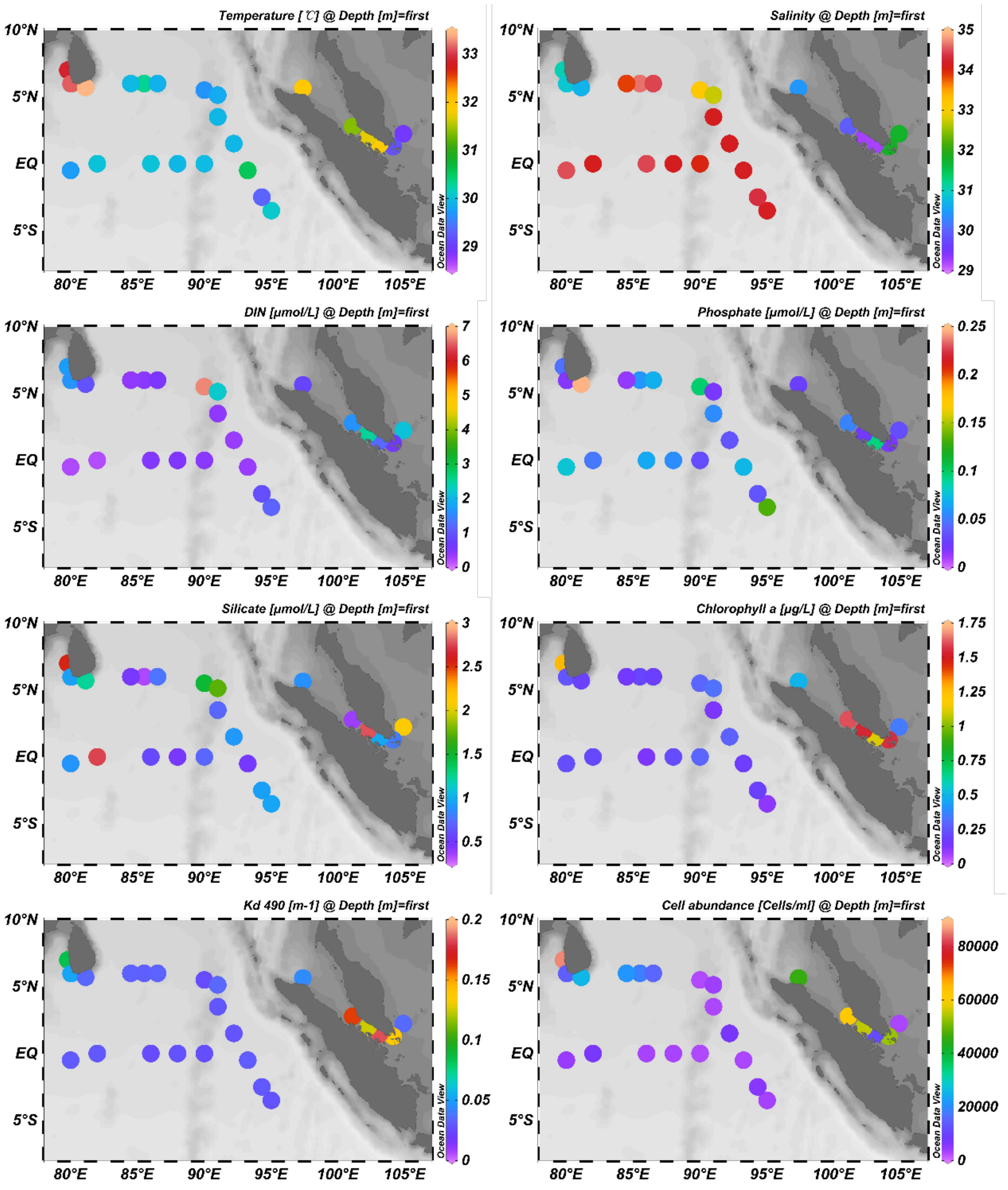

Fig. S1. The distribution of temperature, salinity, dissolved inorganic nitrogen (DIN), phosphate, silicate, chlorophyll *a* concentration, Kd490 (the diffuse attenuation coefficients at 490 nm), and *Synechococcus* cell abundance in the surface water of eatern Indian Ocean (EIO), coastal waters of Sri Lanka and Strait of Malacca.

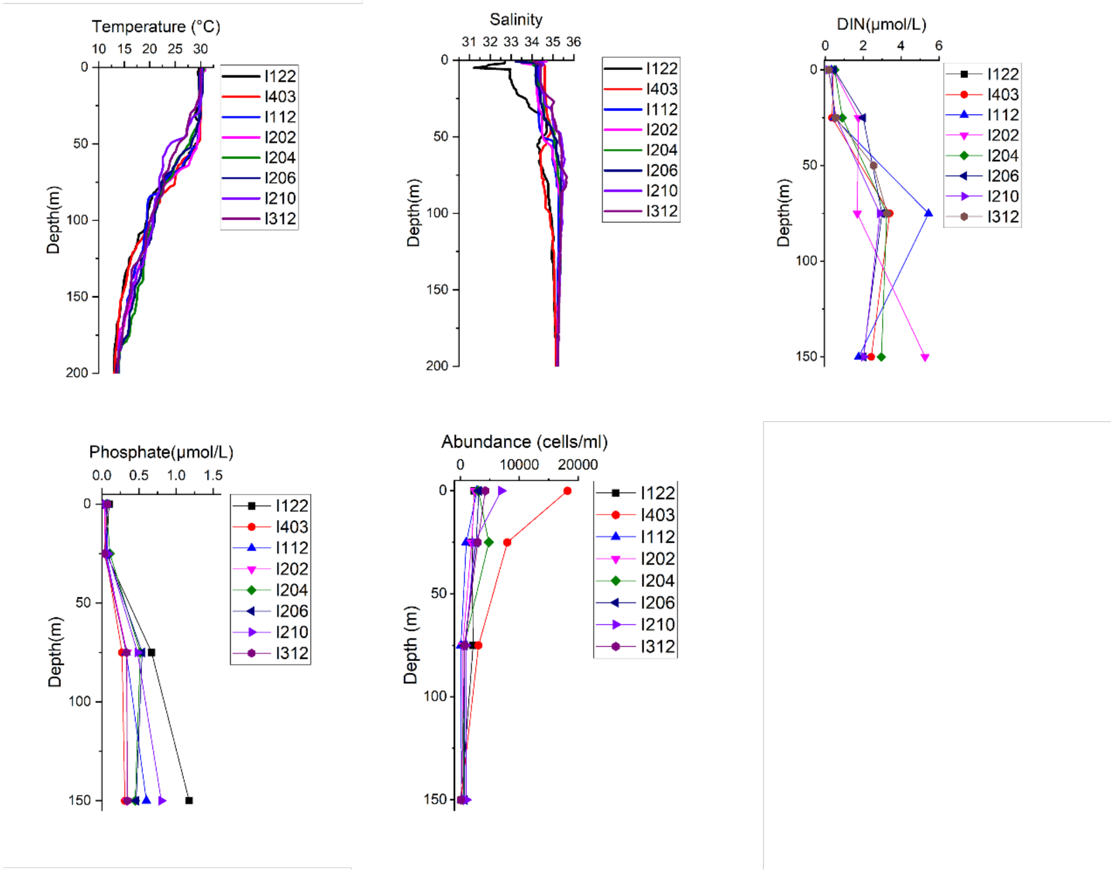

Fig. S2. The vertical profile of temperature, salinity, dissolved inorganic nitrogen (DIN), phosphate and *Synechococcus* cell abundance at sampling stations in eatern Indian Ocean (EIO).

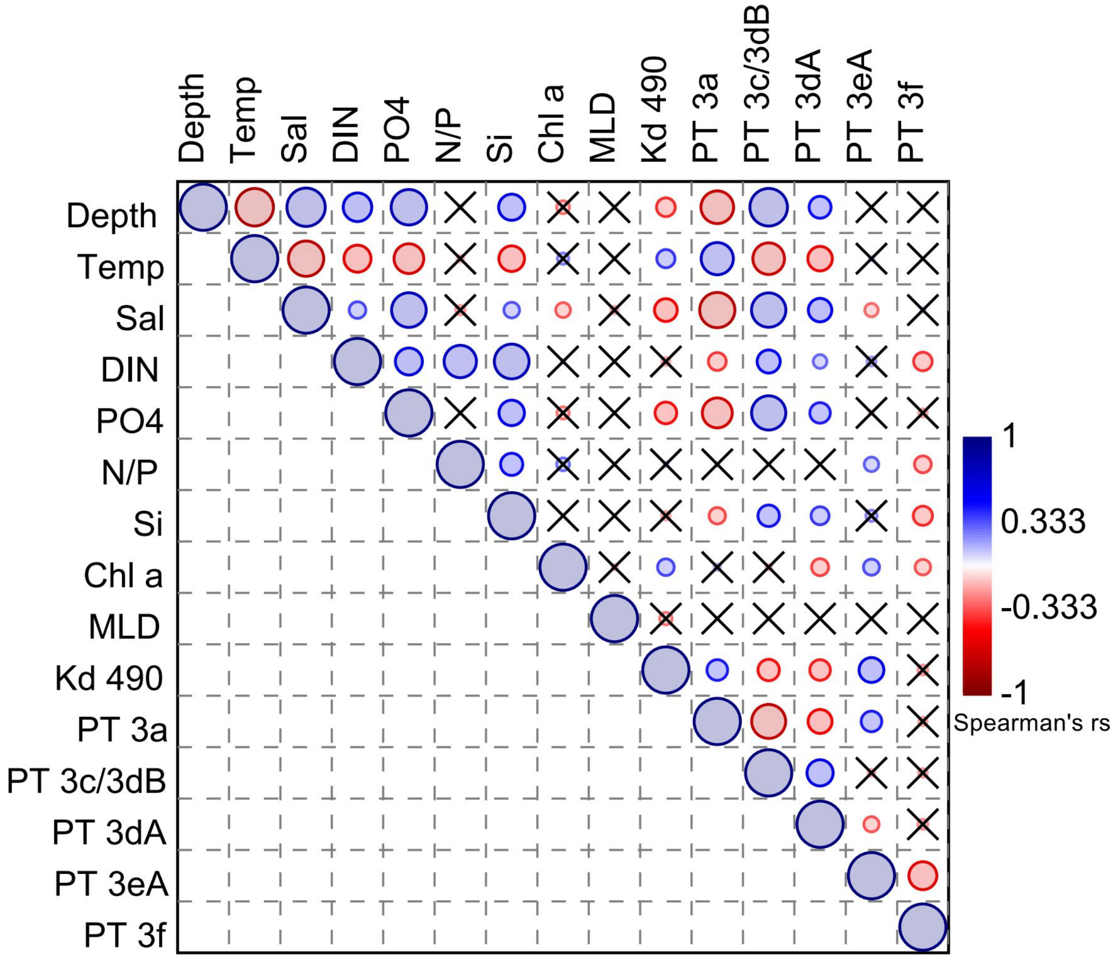

Fig. S3. Correlation analysis between *Synechococcus* pigment types and environmental parameters based on Spearman analysis. The circle scale shows the degree of postive correlation (blue) or negative correlation (red) between two variables and a corss means the correlation was not significant (p>0.05). Abbreviation in the figures: Temp, temperature; Sal, salinity; DIN, dissolved organic notrogen; PO4, phosphate; N/P, nitrogen/phosphate ratio; Si, silicate; Chl *a*, chlorophyll *a*; MLD, mixed layer depth; Kd490, the diffuse attenuation coefficients at 490 nm.

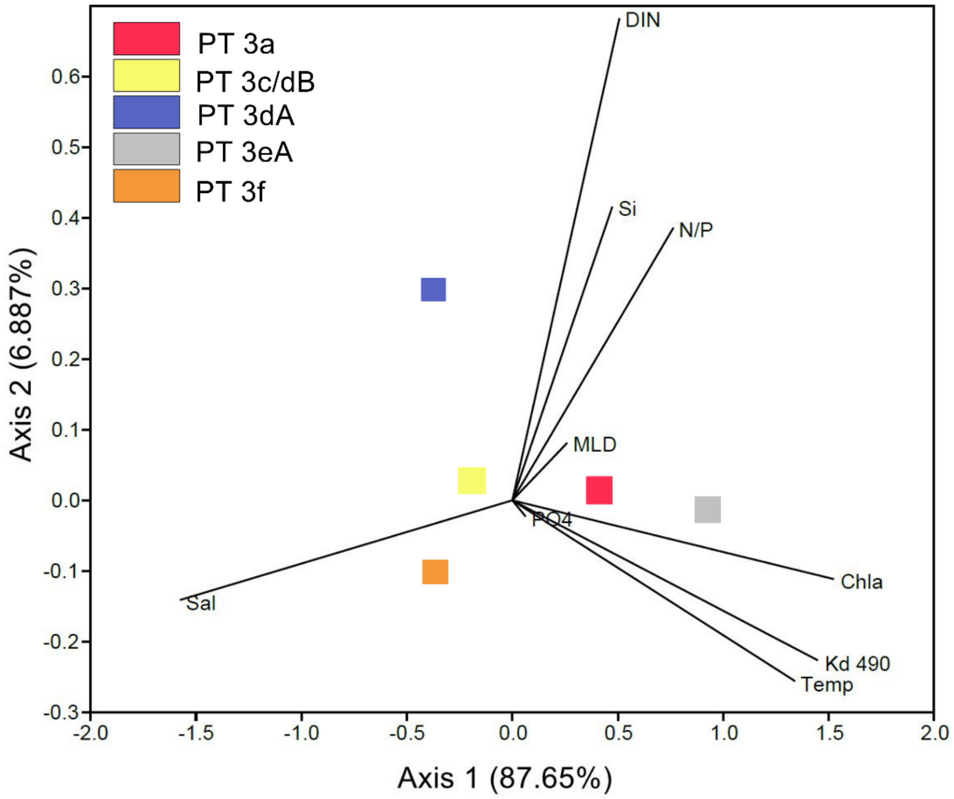
PT 3a
PT 3c/dB
PT 3dA
PT 3eA
PT 3f
DIN
Si
N/P
MLD
PO4
Chla
Sal
Kd 490
Temp
Axis 2 (6.887%)
Axis 1 (87.65%)

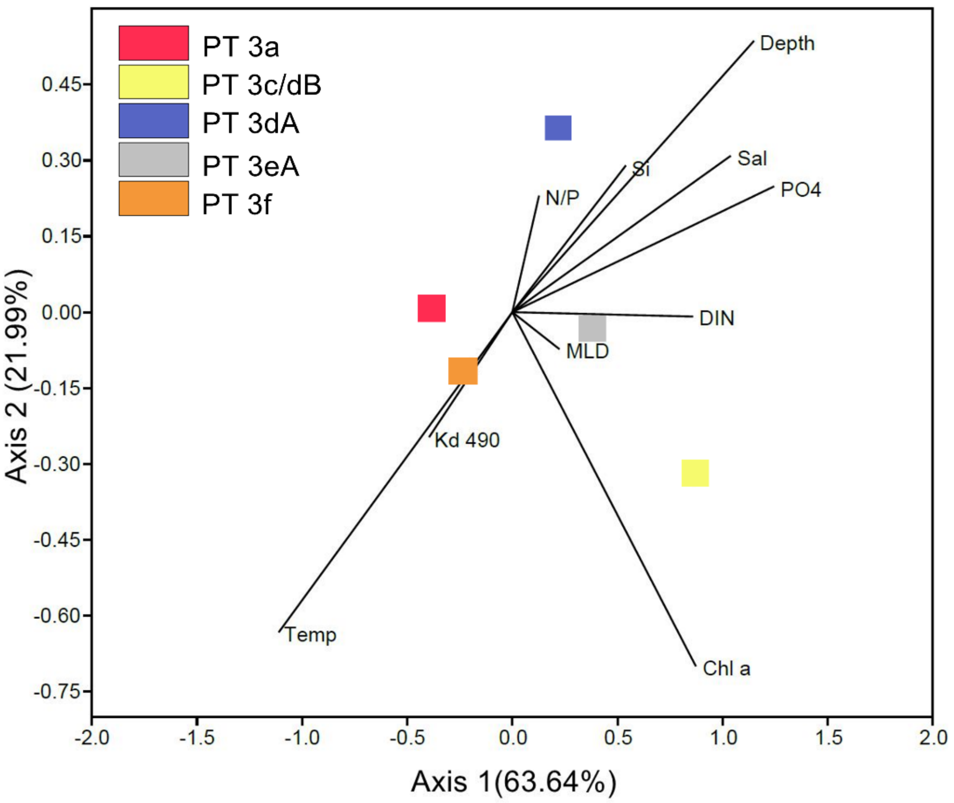
PT 3a
PT 3c/dB
PT 3dA
PT 3eA
PT 3f
Depth
Sal
Si
PO4
N/P
DIN
MLD
Kd 490
Temp
Chl a
Axis 2 (21.99%)
Axis 1(63.64%)

Fig. S4. CCA analysis of community of *Synechococcus* pigment types in surface waters and water column of sampling stations. Abbreviation in the figures: Temp, temperature; Sal, salinity; DIN, dissolved organic notrogen; PO4, phosphate; N/P, nitrogen/phosphate ratio; Si, silicate; Chl *a*, chlorophyll *a*; MLD, mixed layer depth; Kd 490, the diffuse attenuation coefficients at 490 nm.

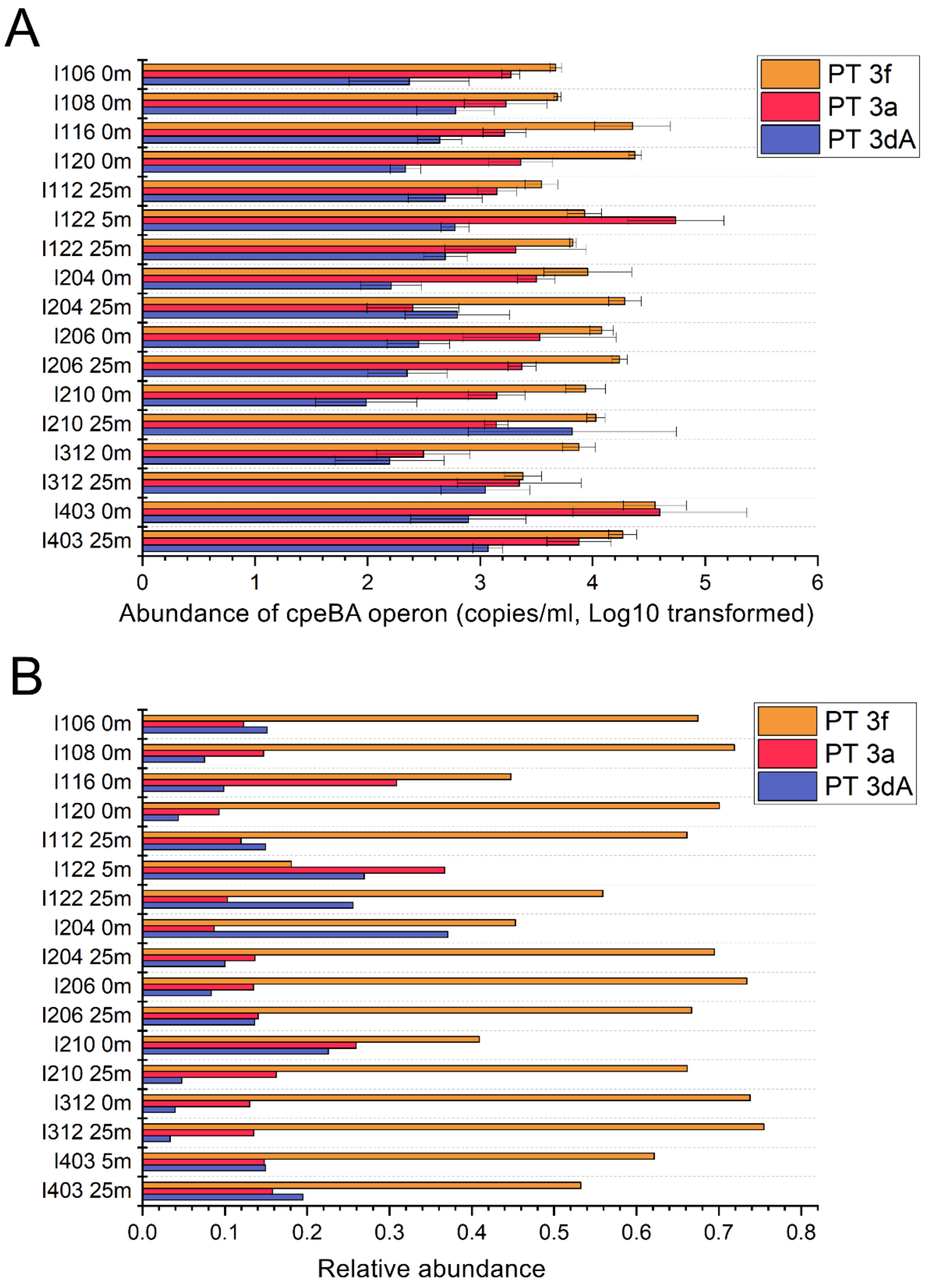

Fig. S5. Comparison of relative abundance and *cpeBA* operon gene copies concentration of PT3a, PT3dA and PT3f in EIO. A: The *cpeBA* operon gene copies concentration (copies/ml, Log10 transformed); B: Relative abundance of targeting OTUs
